# Development and Validation of a Multi-Dimensional Measure of Activity-Based Working Behaviors

**DOI:** 10.3389/fpsyg.2021.655881

**Published:** 2021-10-20

**Authors:** Gisela Bäcklander, Rebecca Fältén, Christina Bodin Danielsson, Susanna Toivanen, Anne Richter

**Affiliations:** ^1^The Swedish School of Sport and Health Sciences, Stockholm, Sweden; ^2^Medical Management Centre, Karolinska Institute, Solna, Sweden; ^3^Department of Psychology, Stockholm University, Stockholm, Sweden; ^4^School of Architecture, KTH Royal Institute of Technology, Stockholm, Sweden; ^5^School of Health, Care, and Social Welfare, Mälardalen University, Västerås, Sweden

**Keywords:** activity-based working (ABW), flex office, scale development and validation, task-environment-fit, office types, proactive work behaviors, activity-based flexible office, crafting behaviors

## Abstract

Most work on activity-based working centers on the physical environment and digital technologies enabling flexible working. While important, we believe the key components for implementing activity-based working are employee and manager behaviors. To measure the degree of enactment of activity-based work, based on workshops with experienced practitioners as well as previous literature, we have developed and validated a behavior-focused measure of activity-based working behaviors. In our initial sample (Sample 1, *N* = 234), three subscales were identified: task – environment crafting, workday planning, and social needs prioritization. In the replication sample (Sample 2, *N* = 434), this model also showed adequate fit. Moreover, task – environment crafting was related to general health and lower stress in sample 1 (multi-organization sample), but not in the single-organization sample (sample 2). Workday planning was associated with higher concentration in both samples and in the second sample with general health and work engagement; the latter was also related to social needs prioritization.

## Introduction

Over the past several decades, increased workplace flexibility has been a trend in the working life of white-collar workers (Stone and Luchetti, 1985; Appel-Meulenbroek, 2016a; Mache et al., 2020). Workplace flexibility is the opportunity to adjust where, when, and how work is performed (Putnam et al., 2014). In line with this, ways of working have changed because information and communication technologies (ICTs) have quickly developed, making it possible to work virtually everywhere and at any time for many employees (Burke and Cooper, 2000; Grant et al., 2010; Allvin et al., 2013).

Activity-based working environments are a flexibility concept centered on the office’s layout, offering a variety of spaces for work such as smaller rooms for concentrated work, quiet zones, open lounge areas, and meeting rooms. In activity-based working environments, employees often do not have fixed seats, are equipped with extensive digital solutions, and have significant discretion over where to work. While they have a historical predecessor in the “non-territorial office” at IBM in the 1970s, their current incarnation was coined in the 1990s by Dutch consulting company Veldhoen (Parker, 2016). Activity-based working environments have become more common in many European and North American organizations (Sivunen and Putnam, 2020). Typical drivers for adopting activity-based offices are to reduce costs, facilitate collaboration between employees but also to position the organization as forward-thinking, change-capable, modern, and innovative to be attractive to skilled talent; and organizational change—specifically so that employees will change their behavior to new ways of working (van der Voordt, 2004; Davis et al., 2012; Kim et al., 2016; Gerards et al., 2018; Rolfö et al., 2018). However, these office types are not uncontroversial. The media has reported numerous anecdotes about negative effects that activity-based working environments might have, and scientific results on this matter are mixed (Bodin Danielsson and Bodin, 2008; De Been and Beijer, 2014; Seddigh et al., 2014; Fagerström, 2016; Jungstedt, 2016; Wilhelmsson, 2016; Backman, 2017; Babapour, 2019; Bäcklander et al., 2019; Rolfö et al., 2019; Wohlers et al., 2019).

When organizations redesign the physical workspace to provide an environment that fosters efficiency and creativity, employees also need to change their work behaviors to make use of that new design. However, this shift in employee behavior is more challenging than management typically expects (Appel-Meulenbroek, 2016b; Hoendervanger et al., 2016). As a result, several scholars have called for increased attention to the implementation process and, in particular, employee behaviors in activity-based working environments (Nielsen, 2013; Appel-Meulenbroek, 2016a; Gerdenitsch et al., 2018). The mixed effects have several potential reasons. For example, for some organizations the concept of activity-based flexible offices (A-FO’s) might not fit well (intervention failure), whereas for others the concept fits but its implementation was conducted poorly (implementation failure). So far, research on A-FOs has primarily focused on understanding its effects on employee well-being or productivity (see for example a recent systematic review that summarized the empirical evidence around the effects of the A-FO; Engelen et al., 2019). However, to fully understand how these effects may arise, it is important to understand what employees do in activity-based working environments. Understanding employee behaviors and how frequently these are performed is an important explanatory factor how effects of the work environment translate into health or productivity relevant for employees and organizations. Furthermore, it helps understand the implementation success of the A-FO which might be another important explanatory factor understanding the effects that the physical work environment may have on employees and organizations.

To build on this endeavor, we detail here the development and validation of a measure of activity-based working focused on employee behaviors in an activity-based office. This study makes both theoretical and practical contributions. First, our measure makes incorporating employee behaviors into research on activity-based offices simpler and thereby offers a clearer way to study implementation and, especially, the success of various implementation strategies. It further enables a clearer and deeper understanding to be reached of the mixed effects of activity-based work environments found in the literature. Conceptualizing activity-based working will also broaden the theoretical understanding of proactive work behavior. From a practical perspective, our measure gives organizations a means of assessing the degree of activity-based working adopted by employees, which may help them to plan intervention strategies, aim these at the relevant issues, and continually work on their implementation.

## Background of the Current Study

### Activity-Based Offices

*Activity-based working environments* is an umbrella term for different office types that put the work activity at the center of the office design. These designs include office types where working locations are *shared*, whether open or enclosed (Bodin Danielsson and Bodin, 2008; De Been and Beijer, 2014). The most frequent office types are called *flex*- and *combi*-offices (Bodin Danielsson et al., 2015). Both office types are activity-based; as such, they offer several types of workstations and environments for both individual and joint working. The difference is that in activity-based flex-offices (A-FOs), employees have no personal workstations (Wohlers and Hertel, 2017) as they do in combi-offices. Today, A-FOs are the most common activity-based office type, which is why we have chosen to investigate A-FOs in this study.

### Effects of the Activity-Based Flexible Office

The effects of A-FOs are mixed. Regarding satisfaction with the physical environments, having access to supportive facilities—like rooms for collaboration or for private work—seems essential for employee satisfaction. Given this, A-FOs fare well in comparison to other office types (Brunia et al., 2016; Bodin Danielsson and Theorell, 2019; Wohlers et al., 2019). Studies have found employee autonomy to be positively related to A-FOs (Vos and van der Voordt, 2002; Wohlers and Hertel, 2017; Bäcklander et al., 2019). Furthermore, from a health perspective, sedentary time has been found to decrease (e.g., Foley et al., 2016), but A-FOs have also been associated with excess risk of absence due to sickness as well as more sick days, for men but not for women (Bodin Danielsson et al., 2014). A-FOs’ effects on employees’ productivity are contradictory. A positive relationship with employees’ informal learning has been found (Gerards et al., 2018), partially mediated by the frequency of feedback from supervisors and co-workers. In line with this, desk-sharing at A-FOs is associated with increased communication (De Croon et al., 2005), higher satisfaction with support of affinity and social interaction in this office type (Bodin Danielsson and Bodin, 2009), positive long-term health effects (Meijer et al., 2009) and improved self-rated productivity and health compared to an open-plan office with fixed workstations (Kim et al., 2016). Yet, other studies do not support these findings. For example, a study from four organizations found that employees in companies that implemented A-FOs reported lower levels of productivity, health, and satisfaction (Appel-Meulenbroek et al., 2011), and face-to-face communication was in one study shown to decrease rather than increase (Bernstein and Turban, 2018).

### Activity-Based Working: A New Way of Working in Activity-Based Flex Offices

Transitioning to an A-FO must be accompanied by new ways of working. Compared to working in a traditional cell office or open plan office with personal desks, working in an A-FO is characterized by high employee discretion over the timing and place of their work, as well as through the management of output, allowing autonomy over how work is conducted (Demerouti et al., 2014; Gerards et al., 2018). The physical work environment in an A-FO shall create the optimal intersection between employee behavior, the virtual and the physical environment (Veldhoen Company, 2021). Employees themselves organize their work in a functional and productive way by seeking out the optimal environmental conditions for each activity in which they engage (e.g., developing ideas, delivering content, or sharing knowledge). Different kinds of environments stimulate and support different types of activities. For example, developing ideas might require a space where concentration is possible, whereas sharing knowledge requires a very different environmental context where it is easy to interact with others. Hence, when employees work activity-based, they do not have an individual personalized workstation but rather chose amongst different environments that support them in their task at hand. Therefore, it is required that employees use workspaces responsibly, for example, remove their working material when the station is no longer needed to make it available to other employees. Besides having different workstations that can be used flexibly, functioning and adapted IT solutions must be in place that allows people to work independent of location and without reliance on manuscript (e.g., apps to locate one’s colleagues, printing on the go, video conferences, organizational knowledge available anywhere, and cloud-based working). Working activity-based is one type of proactive work behavior, where employees actively change their work environment (cf. Frese et al., 2007). The actions (e.g., choosing workplace) are future-focused as well as self-directed and aim at bringing about change to the direct physical work environment (Bindl and Parker, 2011). A higher level of proactivity is needed as the regular presence of a supervisor or of one’s colleagues is not guaranteed to guide work. Moreover, trust and self-management are part of the activity-based way (van Koetsveld and Kamperman, 2011). Job crafting, the proactive behavior for modifying job tasks, the meaning of work or relationships at work (Wrzesniewski and Dutton, 2001), seems a related construct to activity-based working. Whilst job crafting focuses primarily on the content and meaning of the job (Tims et al., 2012; Slemp and Vella-Brodrick, 2013), activity-based working focuses solely on the process of how and where work is conducted. Both represent constructs that aim at increasing fit: job crafting focuses on the person-job fit (Tims et al., 2016; Kooij et al., 2017), whereas we argue that activity-based working aims at increasing the job-environment fit. Planning work ahead of time further facilitates implementing behavior (Gollwitzer, 1999), in this case matching the environment to the planned task. Planning is relevant to coordinate people, documents, or access to technology, possibly booking rooms, and other actions that may be needed in relation to tasks and environments.

Hence, introducing an A-FO in an organization does not only require the physical office to be redesigned or the IT solutions to be upgraded. Employees need to work in an activity-based way to be able to utilize the A-FO environment to the fullest and to reach the intended outcomes (e.g., increased collaboration; Veldhoen Company, 2021). That means personnel in the organization need to make a behavioral change, from a territorial to an activity-based way of working in this new office (cf. Michie et al., 2014). Understanding the A-FO and its effects also requires a deeper understanding of employee behaviors in this new office space. Relating to intervention and implementation research, one important step for evaluation is to measure the target behavior (in our case, the activity-based working) and understand if and in which direction employee behavioral change has happened (Pawson and Tilley, 1997; Michie et al., 2014). Achieving a behavioral change at all levels in the organization (e.g., among managers, support staff, and employees) is key to any organizational change but is often neglected in favor of a focus on artifacts—the “things” of a change—such as the physical office design in an A-FO setting (Pentland and Feldman, 2008; Nielsen, 2013). Hence behavioral change is often not measured. Furthermore, activity-based work environments in particular are not strongly designed situations (i.e., unambiguously signaling how to behave; Mischel, 1977) because the constellations of employees and places are fluid, and the concept relies on trust, autonomy, and self-management (van Koetsveld and Kamperman, 2011). Employees have considerable freedom of action, which also means that they must continually enact new ways of working to realize the concept. So far, few attempts have been made to operationalize activity-based *working behaviors* in an A-FO, and the question arises as to whether the type of working environment (that is, the A-FO) or the implementation of the A-FO and activity-based working (that is, the actions to bring about this organizational change) is actually the reason for the observed mixed effects of A-FOs. For example, realizing that employees are not working activity-based (e.g., not changing work station based on the task, instead sit in the same place independent of the task and block this workstation for others) – this might not only be a less than optimal work environment for the individual’s work tasks (e.g., sitting in a collaboration zone where many discussion are ongoing while working on a task that requires high levels of concentration) but may create conflicts between employees who use the A-FO as intended and employees who display more territorial behaviors, for example. Disentangling such effects is a common difficulty of field studies, but the knowledge gained from field studies can be increased by measuring the implementation process more closely and with finer granularity (e.g., by operationalizing activity-based working; Lipsey and Cordray, 2000). To facilitate finer granularity in collecting data on the implementation process of ABW as well as to facilitate evaluations of A-FOs, we developed the activity-based working behaviors (ABW-B) scale, based on co-creating and an inductive methodology with practitioners, which we validated with two quantitative samples.

The intended mode of working in A-FOs entails making use of the space and the digital technologies, and employees choose where to work based on what environment (in the broadest sense) will support the work one intends to do. The idea of ABW is to plan and proactively choose the environment suitable for the work and thus craft task–environment fit. Our ABW-B scale operationalizes these target behaviors, which are the core enactment of ABW. Therefore, the overall aim of this study is to develop and validate an activity-based working scale, the ABW-B. Moreover, we investigate how the ABW-B is related to well-being (health, stress, concentration, and work engagement).

## Materials and Methods

### Item Development and Evaluation of the Scale

**FIGURE 1:**
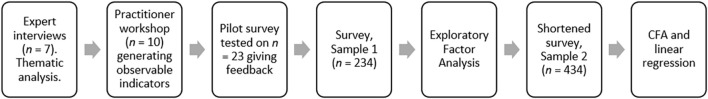
Development and analysis process.

An overview of the steps of the development process is given in Figure 1. Items were generated based on two sources. First, seven semi-structured interviews were conducted to identify characteristics of working in an A-FO (for the interview guide, see Appendix A). Participants were selected based on their expertise in A-FOs. Beyond being provided with verbal and written information about the project’s aim, the participants were informed that there were no right or wrong answers to the questions and that their participation was voluntary before they gave written consent. The interviews lasted up to 50 min and were audiotaped and transcribed verbatim. Based on a thematic analysis, four overall themes emerged (implementation process, ways of working, physical environment, and leadership), with 11 subthemes. In this manuscript, we detail the development of survey measures based on the *Ways of Working theme*, i.e., the employee behavioral component. We consider as out of scope for this manuscript those themes and items relating to supportive factors, not least since these have more overlap with existing measures, such as supervisor support (as a part of the leadership theme). Table 1 details the Ways of Working theme and subthemes, with illustrative quotes.

**TABLE 1:** Subthemes and illustrative quotes for master theme II, Ways of Working, feeding into the activity-based working behaviors (ABW-B) subfactors.

Theme	Sub themes	Examples of sub themes
(1) Social relations	(a) Create space and a routine for socialising	(A) Always have “monday-fika” over a videoconference, or have a space at the office where everyone meets at specific times IP1: You have to decide to meet. Because you have the possibility to disappear IP7: “Us humans often want to sit with our friends. We want our territory closer to the group. So, there is a strong drive to sit more with the same people, the same voices, those you like.” (a not entirely positive example)
(2) Office rules	(a) Guidelines for how to work	(A) Always leave clean desk when you leave IP4: “Some people find their favourite spot and want to sit there all the time. Their mode of working does not always correspond to the activity the zone is meant for” IP7: (comparing with non-ABW) “You know where to sit, you don’t need to plan… you don’t need to take your things, you can keep them on the desk. In the activity-based office you need to make choices. So when you come in the morning you need to plan a little, what I will do that day”
(3) Self-leadership	(a) Plan your own work (b) Responsibility	(A) A-FO requires more planning, what do I do this week/today? Where do I sit for different tasks? What type of zone is suitable? What equipment do I need? Book meeting rooms IP3: “‘I have a meeting at two and quick touch base at ten.’ Ok, so, what are my work tasks? And this is the difficult part, because now I have to take responsibility and plan my day. I have to be mentally present and decide, why am I doing something? What will I do? How? When? With whom? And in the activity-based workplace - where?” IP5: “You have to have thought about your day. ‘Now I will do this or that, and that is supported in this kind of environment’ so I go to a meeting room or a quiet room or a team zone. So you need to plan your workday to be productive” (B) The responsibility for the work is often moved from the manager/team to employees IP7: “You have to make conscious choices. /…/ If you are a person who is used to work with given tasks and maybe don’t have to take responsibility for the whole – because there are still people like that in our work lives – they may not like this lack of predictability and not being able to plop down at your desk and start working with whatever happens to be closest”
(4) Digital work	(a) IT-solution	Proper IT-solutions that suit the organization. Educate everyone and create guidelines for how to use the digital equipment, so that they are used
(5) Iterative work	(a) Continuously change and adopt	The company grows or shrinks. There are changed or new demands or inquire which requires changes in the company. Flexible setting at the office.

The next step was a workshop with 10 practitioners from four organizations working with or having an A-FO was held to identify crucial employee behaviors specific to working in an A-FO. The co-created program logic methodology (described in von Thiele Schwarz et al., 2018) was used for structured brainstorming. The participants individually identified important behaviors when working in A-FOs and documented them on sticky notes. Example sticky notes are: “in activity-based work, the employee follows the work,” “employees need to find out where colleagues are,” and “employees don’t leave their things at the desk.” Next, the participants categorized the sticky notes based on common themes, first individually and then as a group.

In the next step, the input from both interviews and the workshop was taken, and 16 items on activity-based working behaviors were generated. These items were tested on 23 employees working in an A-FO setting and provided feedback on the understandability of the items as well as on the content of the items. Moreover, the participants could add behaviors crucial to ABW that were not captured by the existing items. Based on their feedback, four items were removed as for example the pilot testing revealed that the phrasing of the items was unclear. Moreover, smaller improvements and revisions to the remaining 12 items were made. This resulted in 12 items capturing employee behaviors in the A-FO that were further statistically evaluated in two samples. Sample 1 was used to first identify a factor structure (Sample 1), which then was tested further in Samples 2.

### Participants

#### Sample 1

Sample 1 was a Swedish convenience sample consisting of participants who were invited to participate in the survey online via the social media platform LinkedIn as well as employees from four organizations having an A-FO who were invited to participate in a paper-and-pencil version. In total, 240 employees responded to the survey (95 online, 145 paper and pencil). After screening for incomplete responses and excluding employees who reported not to work in an A-FO, *N* = 234 answers were used for further analysis. The participants’ age ranged from 23 to 65 years, with a mean of 42 years (*SD* = 10.1). Furthermore, 50% of the participants were women, and the majority (79.5 %) of participants had 3 years or more of postsecondary education. On average, the participants had worked in an A-FO for two years, but the median was 10 months.

#### Sample 2

Sample 2 was gathered from 1,344 employees from one Swedish organization, which had relocated to an A-FO 1 year before the data collection. In total, 434 responded with valid information on the A-FO items (32.3% response rate); of these respondents, 81 (18.7%) were managers. The participants’ age ranged from 24 to 65 years, with a mean of 43.8 years (*SD* = 9.1). Of the participants, 59.3% were women, and the majority (67.8%) had three years or more of postsecondary education. The vast majority (99.5%) of the respondents had a permanent contract and worked full time (97%). Their tenure ranged between 0 and 39 years, with mean = 7.3 years (*SD* = 7.5) and median = 5 years.

### Measures

*General health* was assessed with a single item (“How would you assess your health?”) and alternatives ranging from 1 to 5 (1 = *very poor* and 5 = *very good*). This measure has been shown to have good predictive validity of mortality (Burström and Fredlund, 2001; DeSalvo et al., 2005). *Stress* was measured with a single item (“How often have you felt stressed in the past few weeks?”; Dallner et al., 2000; Elo et al., 2003). Answers ranged from 1 to 5 (1 = *Every day* and 5 = *Less than once a month*, *or never*). *Concentration troubles* were measured using three items from the COPSOQ cognitive stress subscale (Kristensen et al., 2005). An example item is: “In the past 2 months, I have had problems concentrating.” Answering alternatives ranged from 1 to 5 (1 = *Never* and 5 = *Very often*). Cronbach’s alpha was 0.81 in Sample 1 and 0.86 for Sample 2. *Work engagement* was measured by three items from the Utrecht Work Engagement Scale (Schaufeli and Bakker, 2003; Schaufeli et al., 2006). An example item was: “I find the work that I do full of meaning and purpose.” The answering alternatives ranged from 1 to 5 (1 = *completely disagree* and 5 = *completely agree*). Work engagement was only measured in Sample 2. Cronbach’s alpha was 0.76. The ABW-B was measured using the items indicated in Table 2, which also provides the reliability values, means, standard deviations, and factor loadings for both samples. The answering alternatives ranged from 1 to 5 (1 = *completely disagree* and 5 = *completely agree*).

**TABLE 2:** Cronbach’s alpha, means, standard deviations, and factor loadings of Samples 1 and 2.

		Sample 1 (*N* = 234)			Sample 2 (*N* = 434)		
	Items	*M*	*SD*	Factor loading (EFA)^*a*^	*M*	*SD*	Factor loading (CFA)
*Task – Environment Crafting* (α_Sample 1_ = 0.80; α_Sample 2_ = 0.76)		3.53	1.06	–	3.53	1.00	–
tec01_1,2_	I choose the work zone that is beneficial to my current work task	3.49	1.34	0.881	3.76	1.12	0.837
tec02_1,2_	I deliberately change places depending on the work task	3.20	1.37	0.651	2.91	1.41	0.809
tec03_1,2_	When I need to focus, I work in a “quiet zone”	3.65	1.40	0.510	3.32	1.57	0.493
tec04_1,2_	I choose a work zone to support me in my work task	3.78	1.28	0.807	4.12	1.07	0.741
*Social needs prioritization* (α_Sample 1_ = 0.84; α_Sample 2_ = 0.87)		3.00	1.23	–	3.74	1.17	–
snp01_1,2_	I actively choose to sit near my colleagues	3.14	1.31	0.850	3.89	1.18	0.880
snp02_1,2_	I choose a work zone so I can sit with my closest co-workers	2.87	1.33	0.850	3.59	1.29	0.881
*Workday planning* (α_Sample 1_ = 0.49; α_Sample 2_ = 0.68)		2.69	1.02	–	3.57	0.82	–
wp00^*b*^_1,2_	I break down my workday into smaller tasks so I can choose the right place of work for each task	2.18	1.28	0.569	2.72	1.39	–
wp01_1,2_	I plan my workday before I begin	3.19	1.22	0.569	3.38	1.08	0.701
wp02_2_	I take a look at my work week so I am aware of what my most important tasks will be during the week	–	–	–	4.17	0.86	0.641
wp03_2_	I try to anticipate possible interruptions during my workday and how to handle them	–	–	–	3.16	1.17	0.626

*^a^Per factor, principal axis analysis.*

*^b^In Sample 2, wp00 was excluded from the factor due to poor fit with the other items.*

Based on previous research, three demographic characteristics were used as control variables. Studies have shown relations between gender and health (Baćak and Ólafsdóttir, 2017), gender and stress (Försäkringskassan, 2018), socioeconomic status and stress (Steptoe et al., 2003), age and stress (Shultz et al., 2010), and age and engagement (Kim and Kang, 2017). Hence, age (in years), gender, and higher education, operationalized as three years or more of postsecondary education (yes/no), were controlled for in the analyses.

### Analyses

In Sample 1, the 12 items were tested for skewness and kurtosis. One item was removed due to a strong similarity with an item that was kept. Three items were removed due to poor variation in answers (e.g., the majority answered “1” for disagree). This left 8 of 12 items. Then, exploratory factor analysis with principal axis factoring was performed. Items with factor loadings < 0.40 were excluded.

Next, the proposed model was tested in Sample 2. Here two new items were added to one of the factors to avoid that one sub-factor was only represented by one item. A confirmatory factor analysis was conducted using full information maximum likelihood (FIML) to validate the ABW-B’s proposed factor structure. Four nested models were compared to identify the best fitting model.

To evaluate model fit, we used the comparative fit index (CFI; Bentler, 1990), the Tucker–Lewis index (TLI; Tucker and Lewis, 1973), the root mean square error of approximation (RMSEA; Steiger, 1990), the standardized root mean square residual (SRMR; Bentler, 1995), and the Akaike information criterion (AIC; Akaike, 1987), in addition to the χ^2^ fit statistic. The following recommendations were followed for evaluating the model fit: for CFI, a value above 0.90 is considered a good fit (Bentler, 1990). For TLI, 0.90 has been suggested as a good fit (Hu and Bentler, 1999). Recommendations for RMSEA suggest that a value below 0.10 indicates adequate fit (Browne and Cudeck, 1992; MacCallum et al., 1996), and for SRMR, values below 0.08 suggest a good fit (Hu and Bentler, 1999). The AIC does not have a cut-off but compares relative fits between models (Akaike, 1987; Kenny, 2015), with a lower AIC indicating a better fit. Similarly, for the chi-square-difference test, the model with the significantly lower chi-square value is the better fitting model (Kenny, 2015).

Lastly, criterion-related validity was examined by performing regression analyses with well-being (general health, stress, concentration troubles, and work engagement) as the outcome in both samples. To account for the nestedness of the data in Sample 2 – employees were nested in workgroups represented by one manager – we used multilevel modeling using restricted maximum likelihood (REML). In our sample of 434, a total of 204 managers, that is, workgroups, were represented by 1–9 employees (*M* = 2.13, *median* = 2). In the regressions, the data were clustered by manager. Predictors were grand mean centered. The background variables age, gender, and higher education were modeled on the individual level and controlled for R was used for all of the analyses, specifically the following packages: *apaTables* for the correlation tables (Stanley, 2018), *lavaan* for confirmatory factor analysis (CFA; Rosseel, 2012), *nFactors* and *Psych* for the exploratory factor analyses (Raiche, 2010; Revelle, 2019), and *lme4* for multilevel modeling and regression (Bates et al., 2015).

## Results

### Factor Structure and Descriptive Statistics

The EFA resulted in three potential sub-factors capturing ABW. Four items were excluded based on low factor loadings. Another two items were removed due to redundancy. This process resulted in eight items, categorized into three sub-factors of the ABW-B: task – environment crafting (TEC), workday planning (WP), and social needs prioritization (SNP) behavior. TEC captures the behaviors related to choosing where to work based on the kind of activity or task to be done, aiming to craft a fit between environment and task. WP represents behaviors related to planning the day, such as determining which tasks to do, at the start of the day. SNP refers to an alternative prioritization when deciding where to work; rather than basing the decision primarily on task, one is basing it first on social considerations. Table 2 shows the means, standard deviations, and factor loadings for the valid items, which varied between 0.51 and 0.88.

Four models were compared to test the dimensionality of the ABW-B scale in Sample 2. In Sample 2, two additional items were added so that three items could capture workday planning. We compared the proposed three-factor structure with two alternate two-factor models and a one-factor model (Table 3). According to the chi-square differences test, the proposed three-factor model fit the data significantly better than the comparison models did, at *p* < 0.001. Taken together, the CFI, SRMR, TLI, RMSEA, and AIC suggested an adequate fit. The factor loadings of the items in the CFA of the three-factor model varied between 0.49 and 0.88.

**TABLE 3:** Goodness of fit indices and Chi-Square difference tests of the sub-factors of the ABW-B, Sample 2.

Model	χ ^2^	d*f*	CFI	TLI	AIC	RMSEA	SRMR	Model comparison	Δχ ^2^ (Δ d*f*)
Model 1: Three-factor	112.320***	24	0.926	0.89	11,140	0.092	0.049	–	–
Model 2a: Two-factor a	276.160***	26	0.792	0.712	11,300	0.149	0.095	1 vs 2a	163.840*** (2)
Model 2b: Two-factor b	494.750***	26	0.61	0.46	11,519	0.204	0.113	1 vs 2b	382.430*** (2)
Model 3:	655.482***	27	0.477	0.303	11,677	0.232	0.138	1 vs 3	543.162*** (3)
One-factor									

***p < 0.001. The three-factor model consists of task – environment crafting (TEC), social needs prioritization (SNP), and workday planning (WP) as separate factors; two-factor model a combines TEC and WP in one factor with SNP as the other; two-factor model b combines TEC and SNP in one factor and WP in the other; and the one-factor model includes SNP (which is negatively correlated with TEC), TEC and WP into one factor.

Bivariate correlations of all the measures in the present study and descriptive statistics are presented in Table 4. Task–Environment crafting was directly correlated with all three well-being outcomes collected in sample 1: general health, *r* = 0.25, *p* < 0.01; concentration troubles, *r* = -0.26, *p* < 0.01; and stress, *r* = -0.22, *p* < 0.01 but not in sample 2. In sample 2, TEC was positively related to work engagement (*r* = 0.11, *p* < 0.05). Workday planning was related to all of the well-being outcomes in sample 1, and in sample 2 with general health, concentration troubles, and work engagement. In both samples, SNP was unrelated to health, concentration, and stress, but in sample 2, SNP was positively related to work engagement, which was not measured in sample 1. In both samples, TEC and SNP were negatively related to each other (*r* = -0.14, *p* < 0.05 in sample 1 and *r* = -0.23, *p* < 0.01 in sample 2), supporting their conceptualization as competing rationales or ways of prioritizing how to work. WP and TEC were positively related, while WP and SNP were unrelated, in both samples. All outcome variables were related to each other in both samples (*r*s ranging from 0.24 to 0.61, *p* < 0.01).

**TABLE 4:** Means, standard deviations, and correlations.

Variable	*M* (*SD*) S1	*M* (*SD*) S2	1	2	3	4	5	6a	6b	7	8	9
(1) Age	42.29 (10.05)	43.83 (9.12)	1	0.08	−0.21**	0.07	−0.20**	–0.06		−0.19**	–0.06	0.01
(2) Gender (male)	50%	40.7%	0.07	1	0.15*	0.02	–0.01	0.11		–0.04	0.07	0.10
(3) Postsecondary education	79.5%	67.8%	−0.20**	0.03	1	0.05	0.02	0.19**		0.12	0.01	–0.03
(4) TEC	3.53 (1.06)	3.53 (1.00)	0.02	–0.03	0.09	1	−0.14*	0.47**		0.25**	−0.26**	−0.22**
(5) SNP	3.00 (1.23)	3.74 (1.17)	−0.15**	0.06	–0.00	−0.23**	1	–0.07		–0.02	0.11	–0.06
(6a) WP (two items)	2.69 (1.02)	–								0.16*	−0.23**	−0.16*
(6b) WP (three items)	–	3.57 (0.82)	0.03	–0.02	–0.01	0.31**	0.04	1				
(7) General health	4.17 (0.83)	4.09 (0.72)	–0.08	–0.01	–0.02	0.07	0.08		0.16**	1	−0.53**	−0.53**
(8) Concentration troubles	2.69 (1.02)	2.52 (0.85)	−0.14**	−0.18**	–0.00	–0.06	–0.02		−0.17**	−0.46**	1	0.59**
(9) Stress	3.58 (1.20)	2.62 (1.18)	0.02	−0.12*	–0.04	0.01	–0.06		–0.01	−0.44**	0.61**	1
(10) Work engagement	–	3.92 (0.75)	0.18**	0.05	–0.06	0.11*	0.20**		0.39**	0.34**	−0.37**	−0.24**

Sample 1 (S1), N = 234, and Sample 2 (S2), N = 434, *p < 0.05; **p < 0.01. The upper triangle is Sample 1’s correlations, and the lower triangle is Sample 2’s correlations. TEC, task – environment crafting; SNP, social need prioritization; WP, workday planning.

### Predictive Validity

In the linear regressions on Sample 1, the predictors were entered in two blocks: as background variables and the ABW-B dimensions. The significance patterns in Block I did not change as Block II was added, and the changes in estimates were minuscule, indicating independence between the background variables and the behavior measures. Because of this, we collapsed the regressions in Table 5 and present estimates only from the version with both blocks. For Sample 2, multilevel regressions were performed with the manager as a clustering variable, and the predictors were similarly entered in two blocks: as background variables and the ABW-B dimensions. As with Sample 1, the significance patterns did not change with Block II, so we collapsed the regressions in the table and present estimates from the full models.

**TABLE 5:** Regression analysis results, β values, and standard errors for Samples 1 and 2.

	General health		Stress		Concentration troubles		Work engagement
Variables	Sample 1	Sample 2^a^	Sample 1	Sample 2^b^	Sample 1	Sample 2^c^	Sample 2^d^
Background							
Age	−0.02**(0.01)	−0.00(0.00)	−0.00(0.00)	−0.00(0.00)	−0.01(0.01)	−0.012*(0.00)	0.02***(0.00)
Gender (male)	−0.07(0.11)	−0.01(0.07)	0.290(0.16)†	−0.284*(0.12)	0.20 (0.13)	−0.292***(0.09)	0.06 (0.07)
Postsecondary educ. ≥ 3 yrs.	0.15 (0.14)	−0.05(0.08)	−0.14(0.20)	−0.03(0.13)	0.00 (0.17)	−0.03(0.09)	−0.04(0.07)
**AB work behaviors**							
TEC	0.225***(0.05)	0.04 (0.04)	−0.19*(0.09)	0.01 (0.07)	-0.13(0.07)†	−0.01(0.04)	0.04 (0.04)
SNP	−0.00(0.04)	0.05(0.03)†	−0.06(0.07)	−0.04(0.05)	0.09 (0.06)	−0.01(0.04)	0.139***(0.03)
WP (two items)	0.01 (0.06)	−	−0.12(0.09)	−	−0.177*(0.08)	−	−
WP (three items)	−	0.13**(0.05)	−	−0.01(0.08)	−	−0.168**(0.05)	0.332***(0.04)
Adj. *R*^2^	0.104***		0.04*		0.078***		
*Conditional R* ^2^		0.042		0.075		0.103	0.236

^†^<0.10; *<0.05; **<0.01; ***<0.001. Sample 2 was subjected to a multilevel analysis with manager as a grouping variable. Sample 2, adjusted ICC: ^a^00; ^b^059; ^c^029; ^d^018. TEC, task – environment crafting; SNP, social need prioritization; WP, workday planning.

#### General Health

In Sample 1, general health was associated with age, β = 0.02, *p* < 0.01, and with task – environment crafting, β = 0.225, *p* < 0.001, meaning those who engaged in more task – environment crafting reported higher levels of health. The model explained about 10% of the variance in health. In Sample 2, general health was associated with workday planning behaviors, β = 0.13, *p* < 0.01, meaning that those doing more planning behaviors reported higher levels of health. The variance explained was about 4%. SNP was not associated with general health in either sample.

#### Stress

For Sample 1, TEC was negatively associated with stress, β = -0.19, *p* < 0.05, with a variance explained of 4%. In Sample 2, only gender was associated with stress, β = -0.284, *p* < 0.05, indicating that women were more stressed than men were. The variance explained was 7.5 %.

#### Concentration Troubles

Concentration troubles were negatively associated with workday planning in both Sample 1, β = -0.177, *p* < 0.05 and Sample 2, β = -0.168, *p* < 0.01, meaning that those who did more workday planning reported lower trouble concentrating. For Sample 2, concentration troubles were also related to age, β = -0.012, *p* < 0.001, and gender, β = -0.294, *p* < 0.001, again indicating women having more concentration troubles. In Sample 1, the model explained about 8% of the variance in concentration troubles, and in Sample 2 about 10%.

#### Work Engagement

Work engagement was only measured in Sample 2, where it was associated with age, β = 0.02, *p* < 0.001, and with SNP and WP, β = 0.139, *p* < 0.01 and β = 0.332, *p* < 0.001, respectively. The model explained about 23% of variance in work engagement.

## Discussion

The aim of the present study was to develop and validate the ABW-B, a short measure of ABW behaviors. Considering the prevalence of implementations of A-FOs, the ABW-B is a useful addition to the toolbox of both researchers and practitioners wishing to study A-FO settings. The psychometric performance of the ABW-B was deemed sufficient. The co-created development process (von Thiele Schwarz et al., 2018), using several rounds of workshops, interviews, and content validation as well as refinements of the instrument (Smith and McCarthy, 1995), is an important strength of our study, ensuring both face and content validity.

We found three dimensions (TEC, SNP, and WP) that capture the central behaviors of working in an A-FO. TEC centers both the behavior of switching workstations, which has previously been identified as “appropriate use” of A-FO’s (Gerdenitsch et al., 2018) *and* the motive for the behavior, i.e., switching *so that* a match between task and environment is achieved. SNP focuses on choosing where to sit in a way that prioritizes social needs. The significance of SNP is a bit more ambiguous. While it is a competing rationale of how an employee should go about their day (centering on whom to work close to rather than what activities to do) and is negatively correlated with TEC, it could also be construed as a kind of fit-crafting in its own right (i.e., it is a style of work fitting the individuals’ motivations), which, in that respect, should be related to positive outcomes – as indeed it is to work engagement. It is also possible that SNP is simply a more important need for more social employees (Schueller, 2012; Seddigh et al., 2016). Social cohesion and a sense of belongingness, however, is something that is sometimes seen to take a hit in implementations of ABW (Blok et al., 2012; Haapakangas et al., 2019; Colenberg et al., 2021) and so, there may be some trade off to being extremely task-oriented that is not entirely positive. WP is a supportive behavior to TEC, taking a proactive stance to think through what the activities are that are to be matched with enabling environments. As individuals have fewer environmental cues about what to be working on, as the presence of specific colleagues or supervisors is not given, they need to take a larger responsibility for initiating structure for the workday. As planning is literally engaging (cognitively) with your work, the finding that they relate is expected.

Unlike traditional office layouts, whether cell offices or landscape offices, activity-based flex-offices require a higher degree of proactivity and pre-thought of employees. WP and TEC are proactive behaviors that are especially relevant in the context of activity-based offices. In general, proactive employee behaviors have been shown to be important for creating person – environment fit, such as for organizational newcomers (Kim et al., 2005), for new students (Deng and Yao, 2020), and in job crafting (van Wingerden et al., 2017; Dubbelt et al., 2019). While job crafting focuses on proactively changing the content and focus of the job (operationalized by items such as “I regularly take on extra tasks even though I do not receive extra salary for them” (Tims et al., 2012) or “I give preference to work tasks that suit my skills or interests” (Slemp and Vella-Brodrick, 2013), the aspect of task – environment fit that is covered in TEC is not captured in existing job crafting scales. The ABW-B scales contribute by providing a measurement aimed at the proactive behaviors creating fit between the physical work environment and task.

The ABW-B scale could be used to help differentiate between groups of employees who, to a large degree, work in activity-based ways and hence make use of the facilitating office design versus those who do not, to understand if these two groups experience different kinds of consequences from working in the A-FO. Moreover, supportive interventions could be provided to specific groups based on these results. This differentiation opens up analyses to investigate the mechanisms linked to ABW, speaking to Nielsen and Miraglia (2017) suggestion to examine localized impacts of interventions—a question of “what works for whom, under what circumstances?” Modern ways of working put demands on workers to be proactive and deliberate, and to plan and to remind themselves, as they deal with work that is more fluid and boundaryless (Hannah et al., 2011; Allvin et al., 2013; Hazy and Uhl-Bien, 2015; Wegman et al., 2018; Stewart et al., 2019). Organizations are less controlling in aspects of designing work and are instead offering a platform or arena for proactive and collaborative employees to orchestrate work by their own initiative. However, offering an A-FO is not enough in itself, as the mixed results of A-FO environments have demonstrated (Wohlers and Hertel, 2017; Manca et al., 2018; Bäcklander et al., 2019).

While the proposed factor structure held between the samples, the subscales of the ABW-B had different relationships with well-being within the different samples. A contributing reason for this could be due to the nature of the samples: Sample 1 is mixed, while Sample 2 is all from a single organization. In Sample 2, there was a greater risk of a systemic bias in the form of confounding variables specific to the organizational context. Even though the organizations in Sample 1 most likely also had organizational particularities, due to the mixed nature of the sample, the effects may be diluted, and the signal-to-noise ratio of employee behavior and outcomes will be better (Donaldson, 2012, p. 258). Leadership, work culture, and available spaces likely play a large part in supporting the adoption of ABW and in ensuring success. Such contextual factors also commonly impact the effects of discretionary behaviors, such as we measure here, on various outcomes (Johns, 2018). Another difference between the samples is also the length of being exposed to an A-FO. Whereas Sample 2 had moved to the A-FO a year prior to the survey, more employees in sample 1 had experienced the A-FO environment for longer, which could contribute to differences between the samples.

The study has several limitations. First, we used self-reported data to assess our constructs, which always risks introducing common-method bias (Podsakoff et al., 2003). However, the constructs ask about individuals’ motives for selecting a place of work, which is difficult to assess in other ways, and about subjective perceptions of well-being, for which self-reports are appropriate (Chan, 2009).

Second, general health and stress were each measured with single-item measures, whose psychometric properties are difficult to assess (Fisher and To, 2012). However, the specific measures used have shown good criterion validity and have frequently been used in previous research (Dallner et al., 2000; Burström and Fredlund, 2001; Elo et al., 2003; DeSalvo et al., 2005). Third, we have practiced opportunistic sampling, which may affect the data’s properties. In Sample 1, we collected data from several organizations and also through online self-selection to fill out the survey. Because of this snowball method of survey distribution, it is impossible to say how many people were “invited” to the survey but chose not to participate. Self-selection may have attracted somewhat more polarized views on activity-based work, such as more positive, more negative, and fewer neutral participants. In Sample 2, we collected data from a single organization. While the factor structure of the scale holds up between samples, the relationships between the scale measures and outcomes differ. However, a future empirical question would be to determine more precisely the role of ABW behaviors in organizational implementation processes and their interaction with other variables.

The ABW-B scale has several practical implications. It is a brief, reliable, and valid measurement of the core employee behaviors in enacting an activity-based way of working. The scale would be useful for researchers studying work in activity-based settings with non-personal workstations and especially for capturing the degree of enactment of the desired behaviors. Are employees actually using the A-FO as intended, and does it matter if they do? Organizations may also want to use the measure to gain information about employee behaviors and reasoning in relation to ongoing changes, to better plan and aim relevant interventions. Because the ABW-B is a brief measure, it can easily slot into recurring employee surveys.

Future research would do well to further examine the relationships of the ABW-B scale with other proactive and adaptive discretionary behaviors, especially other fit-creating behaviors such as job crafting (Wrzesniewski and Dutton, 2001; Demerouti et al., 2014). It seems probable that they tap into a general capacity for proactively creating or affecting one’s own working conditions that is especially valuable in a fluid and flexible working life. Although the present study showed different direct relationships with stress, we believe that further examinations of these kinds of continuous or recurring fit-creating behaviors at the micro-level are warranted because, theoretically, they seem to be a resource-demanding way of working. For example, are ABW behaviors to be considered the initiation of effortful self-control and therefore likely more resource-intensive (Gillebaart, 2018), or are they more likely to be less demanding because they focus on regulating behavior through situation selection rather than on dealing with the friction of working in a poorly matched office environment (Duckworth et al., 2016)? Do ABW behaviors help foster goal-setting behaviors at an individual level, leading to higher performance? Future research could also examine relationships with possible antecedents of performing ABW behaviors, like implementation leadership (Mosson et al., 2018) and team climates (Anderson and West, 1998; Mache et al., 2020). Another important research venue is to incorporate the ABW-B scale when studying the implementation of A-FOs. The ABW-B scale indicates if changes in behavior might have happened through a re-localization to an A-FO. Not only is the ABW-B the primary proximal outcome of introducing an A-FO environment, but it can also be used as an important explanatory mechanism to understand potential effects of the A-FO.

## Data Availability Statement

The datasets presented in this article are not readily available because participating organizations have not chosen to share data publicly. Requests to access the datasets should be directed to GB, gisela.backlander@ki.se.

## Ethics Statement

The studies involving human participants were reviewed and approved by Etikprövningsmyndigheten, Swedish Ethical Review Authority. The patients/participants provided their written informed consent to participate in this study.

## Author Contributions

GB: conceptualization, data curation, formal analysis, investigation, methodology, software, validation, and writing – original draft, review, and editing. RF: formal analysis, investigation, methodology, and writing – review and editing. CBD and ST: conceptualization, and writing – review and editing. AR: conceptualization, funding acquisition, project administration, investigation, methodology, supervision, validation, and writing – review and editing. All authors contributed to the article and approved the submitted version.

## Conflict of Interest

The authors declare that the research was conducted in the absence of any commercial or financial relationships that could be construed as a potential conflict of interest.

## Publisher’s Note

All claims expressed in this article are solely those of the authors and do not necessarily represent those of their affiliated organizations, or those of the publisher, the editors and the reviewers. Any product that may be evaluated in this article, or claim that may be made by its manufacturer, is not guaranteed or endorsed by the publisher.

## Appendix A

### Interview Guide, Translated From Swedish

*We want to answer the question:*

*What do you think characterizes work in an activity-based office?*

a. *For ordinary co-workers*
b. *For managers*

*The purpose of this conversation is to better understand what it is like to work in an activity-based office, both from an employee and a manager perspective. We want to understand what employees and managers do differently in the activity-based office compared to other kinds of offices (e.g., cell office or landscape office).*

*We are interested in taking part of experiences and learnings, and theoretical knowledge about the ways of working used in activity-based offices. So, there are no right and wrong answers. The interview will take about 30 min, and you can end the interview at any time. Your answers will be anonymized. <Double check that it is ok that we record the conversation.><Ask the participant to sign the consent form and sign it yourself.>*

1. **Introduction (warm up)**
  - How would you define an activity-based office?
  - Describe your experiences of working in an activity-based office.
2. **Ways of working for employees→ on a behavior level. Imagine someone was being filmed working – what are they doing? As concretely as possible.**
  - As employee: what should you be doing in an activity-based office to use it in the best way?
  - What do employees do differently in an activity-based office compared to a cell or landscape office?
  - What are the five most important things you should do as an employee in an activity-based office for it to work optimally?
  - What common “mistakes” have you seen employees do in an activity-based office? What do employees do when it’s not working? What should you *not* do in an activity-based office?
3. **Ways of working for managers→ on a behavior level. Imagine someone was being filmed working – what are they doing? As concretely as possible.** As a manager: What should you do in an activity-based office to create a good working environment for employees? Think especially of things a manager needs to do differently in an activity-based office compared to a cell or landscape office.
  - What are the five most important things you should do as a manager in an activity-based office for it to work optimally?
  - What common “mistakes” have you seen managers do in an activity-based office? What do managers do when it’s not working?
4. **Finish**
  - I am finished with my questions, is there something you think we missed that you would like to add?
5. **Other interview participants**
  - Could you recommend someone else we should interview about these questions?

## Appendix B

Activity-based Working Behavior Scale (ABW-B), English translation. (1–5; 1 = completely disagree; 5 = completely agree)

**Table:**

*Task – Environment Crafting*	
tec01	I choose the work zone that is beneficial to my current work task.
tec02	I deliberately change places depending on the work task.
tec03	When I need to focus, I work in a “quiet zone.”
tec04	I choose a work zone to support me in my work task.
*Social Needs Prioritization*	
snp01	I actively choose to sit near my colleagues.
snp02	I choose a work zone so I can sit with my closest co-workers.
*Workday Planning*	
wp01	I plan my workday before I begin.
wp02	I take a look at my work week so I am aware of what my most important tasks will be during the week.
wp03	I try to anticipate possible interruptions during my workday and how to handle them.

Activity-based Working Behavior Scale (ABW-B), Swedish original. (1–5; 1 = stämmerintealls; 5 = stämmerhelt)

**Table:**

*Uppgift-Miljö matchning*	
tec01	Jag väljer den arbetszonsomfrämjar min nuvarande arbetsuppgift
tec02	Jag bytermedvetet plats beroendepåarbetsuppgift
tec03	När jag behöver koncentrera mig arbetar jag i en “tystzon”
tec04	Jag väljer en arbetszon som stödjer mig i min arbetsuppgift
*Prioriterar sociala behov*	
snp01	Jag väljer aktivt att sätta mig nära mina kollegor
snp02	Jag väljer arbetszon såatt jag sitter med mina närmaste kollegor
*Planerar arbetsdag*	
wp01	Jag planerar mina arbetsdagar innan de påbörjas
wp02	Jag seröver min arbetsvecka såatt jag vet vad mitt viktigaste arbeteär under veckan.
wp03	Jag försöker att på förhand förutse möjliga avbrott under arbetsdagen och hur jag ska hantera dem.

## References

[B1] Akaike H. (1987). Factor analysis and AIC. *Psychometrika* 52 317–332. 10.1007/BF02294359

[B2] Allvin M. Mellner C. Movitz F. Aronsson G. (2013). The diffusion of flexibility: estimating the incidence of low-regulated working conditions. *Nordic J. Work. Life Stud.* 3 99–116. 10.19154/njwls.v3i3.3013

[B3] Anderson N. R. West M. A. (1998). Measuring climate for work group innovation: development and validation of the team climate inventory. *J. Organiz. Behav.* 19 235–258. 10.1002/(SICI)1099-1379(199805)19:3<235::AID-JOB837<3.0.CO;2-C

[B4] Appel-Meulenbroek R. (2016a). Editorial: Employee behaviour and effects of modern (activity-based) offices. *J. Corp. Real Estate* 18 162–163. 10.1108/JCRE-04-2016-0019

[B5] Appel-Meulenbroek R. (2016b). Modern offices and new ways of working studied in more detail. *J. Corp. Real Estate* 18 2–3. 10.1108/JCRE-02-2016-0010

[B6] Appel-Meulenbroek R. Groenen P. Janssen I. (2011). An end-user’s perspective on activity-based office concepts. *J. Corp. Real Estate* 13 122–135. 10.1108/14630011111136830

[B7] Babapour M. (2019). From fading novelty effects to emergent appreciation of Activity-based Flexible Offices: Comparing the individual, organisational and spatial adaptations in two case organisations. *Appl. Ergonom.* 81:102877. 10.1016/j.apergo.2019.102877 31422254

[B8] Baćak V. Ólafsdóttir S. (2017). Gender and validity of self-rated health in nineteen European countries. *Scand. J. Public Health* 45 647–653. 10.1177/1403494817717405 28673121

[B9] Bäcklander G. Rosengren C. Falkman L. L. Stenfors C. Seddigh A. Osika W. (2019). Navigating the activity-based working environment – relationships of self-leadership, autonomy and information richness with cognitive stress and performance. *Scand. J. Work Organiz. Psychol.* 4 1–14. 10.16993/sjwop.58

[B10] Backman M. (2017). *Vågar du lämna ditt skrivbord (Do you dare leave your desk).* Gothenburg: Göteborgs Posten.

[B11] Bates D. Mächler M. Bolker B. M. Walker S. C. (2015). Fitting linear mixed-effects models using lme4. *J. Stat. Software* 67 1–48. 10.18637/jss.v067.i01

[B12] Bentler P. M. (1990). Comparative fit indexes in structural models. *Psychol. Bull.* 107 238–246. 10.1037/0033-2909.107.2.238 2320703

[B13] Bentler P. M. (1995). *EQS structural equations program manual.* Encino, CA: Multivariate Software, Inc.

[B14] Bernstein E. S. Turban S. (2018). The impact of the “open” workspace on human collaboration. *Philos. Trans. R. Soc. Lond. Ser. B* 373:20170239. 10.1098/rstb.2017.0239 29967303 PMC6030579

[B15] Bindl U. K. Parker S. K. (2011). “Proactive work behavior: Forward-thinking and change-oriented action in organizations,” in *APA handbook of industrial and organizational psychology, Vol 2: Selecting and developing members for the organization*, ed. Zedeck S. (Washington, D.C: American Psychological Association), 567–598. 10.1037/12170-019

[B16] Blok M. M. Groenesteijn L. Schelvis R. Vink P. (2012). New ways of working: does flexibility in time and location of work change work behavior and affect business outcomes? *Work* 41 2605–2610. 10.3233/WOR-2012-1028-2605 22317114

[B17] Bodin Danielsson C. Bodin L. (2008). Office type in relation to health, well-being, and job satisfaction among employees. *Environ. Behav.* 40 636–668. 10.1177/0013916507307459

[B18] Bodin Danielsson C. Bodin L. (2009). Difference in satisfaction with office environment among employees in different office types. *J. Arch. Plan. Res.* 26 241–257.

[B19] Bodin Danielsson C. Theorell T. (2019). Office employees’ perception of workspace contribution: A gender and office design perspective. *Environ. Behav.* 51 995–1026. 10.1177/0013916518759146

[B20] Bodin Danielsson C. Bodin L. Wulff C. Theorell T. (2015). The relation between office type and workplace conflict: A gender and noise perspective. *J. Environ. Psychol.* 42 161–171. 10.1016/j.jenvp.2015.04.004

[B21] Bodin Danielsson C. Chungkham H. S. Wulff C. Westerlund H. (2014). Office design’s impact on sick leave rates. *Ergonomics* 57 139–147. 10.1080/00140139.2013.871064 24460745

[B22] Browne M. W. Cudeck R. (1992). Alternative ways of assessing model fit. *Sociol. Methods Res.* 21 230–258. 10.1177/0049124192021002005

[B23] Brunia S. De Been I. van der Voordt T. J. M. (2016). Accommodating new ways of working: lessons from best practices and worst cases. *J. Corp. Real Estate* 18 30–47. 10.1108/JCRE-10-2015-0028

[B24] Burke R. J. Cooper C. L. (2000). *The organization in crisis: Downsizing, restructuring, and privatization.* Hoboken, NJ: Blackwell Publishers.

[B25] Burström B. Fredlund P. (2001). Self rated health: Is it as good a predictor of subsequent mortality among adults in lower as well as in higher social classes? *J. Epidemiol. Commun. Health* 55 836–840. 10.1136/jech.55.11.836 11604441 PMC1763304

[B26] Chan D. (2009). “So why ask me? Are self-report data really that bad,” in *Statistical and Methodological Myths and Urban Legends: Doctrine, Verity and fable in the organizational and social sciences*, eds Lance C. E. Vandenberg R. J. (Milton Park: Routledge), 309–336.

[B27] Colenberg S. Appel-Meulenbroek R. Romero Herrera N. Keyson D. (2021). Conceptualizing social well-being in activity-based offices. *J. Manage. Psychol.* 36:327343. 10.1108/JMP-09-2019-0529

[B28] Dallner M. Lindström K. Elo A.-L. Skogstad A. Gamberale F. Hottinen V. (2000). *Användarmanual för QPS Nordic: frågeformulär om psykologiska och sociala faktorer i arbetslivet utprovat i Danmark, Finland, Norge och Sverige*, Vol. 19. Stockholm: Arbetslivsrapport.

[B29] Davis M. C. Leach D. J. Clegg C. W. (2012). “The Physical Environment of the Office: Contemporary and Emerging Issues,” in *International Review of Industrial and Organizational Psychology 2011*, Vol. 26 eds Hodgkinson G. P. Ford J. K. (Hoboken, NJ: John Wiley & Sons, Ltd), 193–237.

[B30] De Been I. Beijer M. (2014). The influence of office type on satisfaction and perceived productivity support. *J. Facil. Manage.* 12 142–157. 10.1108/JFM-02-2013-0011

[B31] De Croon E. Sluiter J. Kuijer P. P. Frings-Dresen M. (2005). The effect of office concepts on worker health and performance: a systematic review of the literature. *Ergonomics* 48 119–134. 10.1080/00140130512331319409 15764312

[B32] Demerouti E. Derks D. ten Brummelhuis L. L. Bakker A. B. (2014). “New Ways of Working: Impact on Working Conditions, Work–Family Balance, and Well-Being,” in *The Impact of ICT on Quality of Working Life*, eds Korunka C. Hoonakker P. (Netherlands: Springer), 123–141. 10.1007/978-94-017-8854-0_8

[B33] Deng Y. Yao X. (2020). Person-environment fit and proactive socialization: Reciprocal relationships in an academic environment. *J. Vocat. Behav.* 120:103446. 10.1016/j.jvb.2020.103446

[B34] DeSalvo K. B. Fan V. S. McDonell M. B. Fihn S. D. (2005). Predicting mortality and healthcare utilization with a single question. *Health Serv. Res.* 40 1234–1246. 10.1111/j.1475-6773.2005.00404.x 16033502 PMC1361190

[B35] Donaldson L. (2012). “Evidence-based management using organizational facts”, in *The Oxford Handbook of Evidence-based Management* ed. Rousseau D. M., Oxford University Press. 10.1093/oxfordhb/9780199763986.013.0014

[B36] Dubbelt L. Demerouti E. Rispens S. (2019). The value of job crafting for work engagement, task performance, and career satisfaction: longitudinal and quasi-experimental evidence. *Eur. J. Work Organiz. Psychol.* 28 300–314. 10.1080/1359432X.2019.1576632

[B37] Duckworth A. L. Gendler T. S. Gross J. J. (2016). Situational strategies for self-control. *Perspect. Psychol. Sci.* 11 35–55. 10.1177/1745691615623247 26817725 PMC4736542

[B38] Elo A. L. Leppänen A. Jahkola A. (2003). Validity of a single-item measure of stress symptoms. *Scand. J. Work Environ. Health* 29 444–451. 10.5271/sjweh.752 14712852

[B39] Engelen L. Chau J. Young S. Mackey M. Jeyapalan D. Bauman A. (2019). Is activity-based working impacting health, work performance and perceptions? A systematic review. *Build. Res. Inform.* 47 468–479. 10.1080/09613218.2018.1440958

[B40] Fagerström E. (2016). Malmös forskare flyr flexkontoren – jobbar hemma Istället. (Malmö’s researchers flee flex offices – work from home instead). Sydsvenskan. Available online at: https://www.sydsvenskan.se/2016-11-27/malmos-forskare-flyr-flexkontoren–jobbar-hemma-istallet

[B41] Fisher C. D. To M. L. (2012). Using experience sampling methodology in organizational behavior. *J. Organiz. Behav.* 33 865–877. 10.1002/job.1803

[B42] Foley B. Engelen L. Gale J. Bauman A. Mackey M. (2016). Sedentary behavior and musculoskeletal discomfort are reduced when office workers trial an activity-based work environment. *J. Occup. Environ. Med.* 58 924–931. 10.1097/JOM.0000000000000828 27454397

[B43] Försäkringskassan (2018). *Sjukfrånvaron på svensk arbetsmarknad (Sick leave in the Swedish job market), 2018:2.* Available Online at: www.forsakringskassan.se (accessed October 04, 2021).

[B44] Frese M. Garst H. Fay D. (2007). Making things happen: Reciprocal relationships between work characteristics and personal initiative in a four-wave longitudinal structural equation model. *J. Appl. Psychol.* 92 1084–1102. 10.1037/0021-9010.92.4.1084 17638467

[B45] Gerards R. de Grip A. Baudewijns C. (2018). Do new ways of working increase work engagement? *Person. Rev.* 47 517–534. 10.1108/PR-02-2017-0050

[B46] Gerdenitsch C. Korunka C. Hertel G. (2018). Need–supply fit in an activity-based flexible office: A longitudinal study during relocation. *Environ. Behav.* 50 273–297. 10.1177/0013916517697766

[B47] Gillebaart M. (2018). The ‘Operational’ definition of self-control. *Front. Psychol.* 9:1231. 10.3389/fpsyg.2018.01231 30072939 PMC6058080

[B48] Gollwitzer P. M. (1999). Implementation intentions: Strong effects of simple plans. *Am. Psychol.* 54 493–503. 10.1037/0003-066X.54.7.493

[B49] Grant A. M. Fried Y. Juillerat T. L. (2010). “Work matters: Job Design in Classic and Contemporary Perspectives,” in *APA handbook of industrial and organizational psychology*, ed. Zedeck S. (Washington, D.C: American Psychological Association), 417–453. 10.1037/12169-013

[B50] Haapakangas A. Hallman D. M. Mathiassen S. E. Jahncke H. (2019). The effects of moving into an activity-based office on communication, social relations and work demands – A controlled intervention with repeated follow-up. *J. Environ. Psychol.* 66:101341. 10.1016/j.jenvp.2019.101341

[B51] Hannah S. T. Lord R. G. Pearce C. L. (2011). Leadership and collective requisite complexity. *Organiz. Psychol. Rev.* 1 215–238. 10.1177/2041386611402116

[B52] Hazy J. K. Uhl-Bien M. (2015). Towards operationalizing complexity leadership: How generative, administrative and community-building leadership practices enact organizational outcomes. *Leadership* 11 79–104. 10.1177/1742715013511483

[B53] Hoendervanger J. G. De Been I. Van Yperen N. W. Mobach M. P. Albers C. J. (2016). Flexibility in use: Switching behaviour and satisfaction in activity-based work environments. *J. Corp. Real Estate* 18 48–62. 10.1108/JCRE-10-2015-0033

[B54] Hu L. T. Bentler P. M. (1999). Cut-off criteria for fit indexes in covariance structure analysis: Conventional criteria versus new alternatives. *Struct. Equat. Model.* 6 1–55. 10.1080/10705519909540118

[B55] Johns G. (2018). Advances in the treatment of context in organizational research. *Annu. Rev. Organiz. Psychol. Organiz. Behav.* 5 21–46. 10.1146/annurev-orgpsych-032117-104406

[B56] Jungstedt C. (2016). *Bankanställda i uppror mot kontorstrenden (Banking employees rebel against office trend).* Stockholm, Sweden: Dagens Industri.

[B57] Kenny D. A. (2015). *Measuring Model Fit.* Available Online at: http://davidakenny.net/cm/fit.htm (accessed December 09, 2019).

[B58] Kim J. Candido C. Thomas L. de Dear R. (2016). Desk ownership in the workplace: The effect of non-territorial working on employee workplace satisfaction, perceived productivity and health. *Build. Environ.* 103 203–214. 10.1016/j.buildenv.2016.04.015

[B59] Kim N. Kang S.-W. (2017). Older and more engaged: The mediating role of age-linked resources on work engagement. *Hum. Res. Manage.* 56 731–746. 10.1002/hrm.21802

[B60] Kim T. Y. Cable D. M. Kim S. P. (2005). Socialization tactics, employee proactivity, and person-organization fit. *J. Appl. Psychol.* 90 232–241. 10.1037/0021-9010.90.2.232 15769234

[B61] Kooij D. T. A. M. van Woerkom M. Wilkenloh J. Dorenbosch L. Denissen J. J. A. (2017). Job crafting towards strengths and interests: The effects of a job crafting intervention on person-job fit and the role of age. *J. Appl. Psychol.* 102 971–981. 10.1037/apl0000194 28277726

[B62] Kristensen T. S. Hannerz H. Høgh A. Borg V. (2005). The copenhagen psychosocial questionnaire - a tool for the assessment and improvement of the psychosocial work environment. *Scand. J. Work Environ. Health* 31 438–449. 10.5271/sjweh.948 16425585

[B63] Lipsey M. W. Cordray D. S. (2000). Evaluation methods for social intervention. *Annu. Rev. Psychol.* 51 345–375. 10.1146/annurev.psych.51.1.345 10751975

[B64] MacCallum R. C. Browne M. W. Sugawara H. M. (1996). Power analysis and determination of sample size for covariance structure modeling. *Psychol. Methods* 1 130–149. 10.1037/1082-989X.1.2.130

[B65] Mache S. Servaty R. Harth V. (2020). Flexible work arrangements in open workspaces and relations to occupational stress, need for recovery and psychological detachment from work. *J. Occup. Med. Toxicol.* 15:5. 10.1186/s12995-020-00258-z 32206078 PMC7083021

[B66] Manca C. Grijalvo M. Palacios M. Kaulio M. (2018). Collaborative workplaces for innovation in service companies: barriers and enablers for supporting new ways of working. *Serv. Bus.* 12 525–550. 10.1007/s11628-017-0359-0

[B67] Meijer E. M. Frings-Dresen M. H. W. Sluiter J. K. (2009). Effects of office innovation on office workers’ health and performance. *Ergonomics* 52 1027–1038. 10.1080/00140130902842752 19606364

[B68] Michie S. Atkins L. West R. (2014). *The Behaviour Change Wheel - A Guide To Designing Interventions.* Sutton: Silverback Publishing.

[B69] Mischel W. (1977). “The interaction of person and situation,” in *Personality at the crossroads: Current issues in interactional psychology*, eds Magnusson D. Endler N. S. (Mahwah, NJ: Lawrence Erlbaum Associates), 333–352.

[B70] Mosson R. Von Thiele Schwarz U. Hasson H. Lundmark R. Richter A. (2018). How do iLead? Validation of a scale measuring active and passive implementation leadership in Swedish healthcare. *BMJ Open* 8:e021992. 10.1136/bmjopen-2018-021992 29961033 PMC6042620

[B71] Nielsen K. (2013). Review article: How can we make organizational interventions work? Employees and line managers as actively crafting interventions. *Hum. Relat.* 66 1029–1050. 10.1177/0018726713477164

[B72] Nielsen K. Miraglia M. (2017). What works for whom in which circumstances? On the need to move beyond the ‘what works?’ question in organizational intervention research. *Hum. Relat.* 70 40–62. 10.1177/0018726716670226

[B73] Parker L. D. (2016). From scientific to activity based office management: a mirage of change. *J. Account. Organiz. Change* 12 177–202. 10.1108/JAOC-01-2015-0007

[B74] Pawson R. Tilley N. (1997). *Realistic evaluation.* Thousand Oaks: Sage.

[B75] Pentland B. T. Feldman M. S. (2008). Designing routines: On the folly of designing artifacts, while hoping for patterns of action. *Inform. Organiz.* 18 235–250. 10.1016/j.infoandorg.2008.08.001

[B76] Podsakoff P. M. MacKenzie S. B. Lee J. Y. Podsakoff N. P. (2003). Common method biases in behavioral research: A critical review of the literature and recommended remedies. *J. Appl. Psychol.* 88 879–903. 10.1037/0021-9010.88.5.879 14516251

[B77] Putnam L. L. Myers K. K. Gailliard B. M. (2014). Examining the tensions in workplace flexibility and exploring options for new directions. *Hum. Relat.* 67 413–440. 10.1177/0018726713495704

[B78] Raiche G. (2010). *an R package for parallel analysis and non graphical solutions to the Cattell scree test.* Available Online at: https://cran.r-project.org/package=nFactors.

[B79] Revelle W. (2019). *psych: Procedures for Psychological, Psychometric, and Personality Research.* Evanston, IL: Northwestern University.

[B80] Rolfö L. Eklund J. Jahncke H. (2018). Perceptions of performance and satisfaction after relocation to an activity-based office. *Ergonomics* 61 644–657. 10.1080/00140139.2017.1398844 29134874

[B81] Rolfö L. Jahncke H. Järvholm L. S. Öhrn M. Babapour M. (2019). Predictors of preference for the activity-based flexible office. *Adv. Intell. Syst. Comp.* 876 547–553. 10.1007/978-3-030-02053-8_83

[B82] Rosseel Y. (2012). Lavaan: An R package for structural equation modeling. *J. Stat. Software* 48 1–36. 10.18637/jss.v048.i02

[B83] Schaufeli W. B. Bakker A. B. (2003). *Test Manual for the Utrecht Work Engagement Scale.* Available Online at: http://www.schaufeli.com (accessed January 07, 2019).

[B84] Schaufeli W. B. Bakker A. B. Salanova M. (2006). The measurement of work engagement with a short questionnaire. *Educ. Psychol. Measure.* 66 701–716. 10.1177/0013164405282471

[B85] Schueller S. M. (2012). Personality fit and positive interventions: extraverted and introverted individuals benefit from different happiness increasing strategies. *Psychology* 03 1166–1173. 10.4236/psych.2012.312a172

[B86] Seddigh A. Berntson E. Bodin Danielson C. Westerlund H. (2014). Concentration requirements modify the effect of office type on indicators of health and performance. *J. Environ. Psychol.* 38 167–174. 10.1016/j.jenvp.2014.01.009

[B87] Seddigh A. Berntson E. Platts L. G. Westerlund H. (2016). Does personality have a different impact on self-rated distraction, job satisfaction, and job performance in different office types? *PLoS One* 11:e155295. 10.1371/journal.pone.0155295 27223898 PMC4880328

[B88] Shultz K. S. Wang M. Crimmins E. M. Fisher G. G. (2010). Age differences in the demand—control model of work stress. *J. Appl. Gerontol.* 29 21–47. 10.1177/0733464809334286 20948986 PMC2952960

[B89] Sivunen A. Putnam L. L. (2020). The dialectics of spatial performances: The interplay of tensions in activity-based organizing. *Hum. Relat.* 73 1129–1156. 10.1177/0018726719857117

[B90] Slemp G. R. Vella-Brodrick D. A. (2013). The job crafting questionnaire: A new scale to measure the extent to which employees engage in job crafting. *Int. J. Wellbeing* 3 126–146.

[B91] Smith G. T. McCarthy D. M. (1995). Methodological considerations in the refinement of clinical assessment instruments. *Psychol. Assess.* 7 300–308. 10.1037/1040-3590.7.3.300

[B92] Stanley D. (2018). *apaTables: Create American Psychological Association (APA) Style Tables.* Available Online at: https://cran.r-project.org/package=apaTables.

[B93] Steiger J. H. (1990). Structural model evaluation and modification: An interval estimation approach. *Multivariate Behav. Res.* 25 173–180. 10.1207/s15327906mbr2502_4 26794479

[B94] Steptoe A. Kunz-Ebrecht S. Owen N. Feldman P. J. Willemsen G. Kirschbaum C. (2003). Socioeconomic status and stress-related biological responses over the working day. *Psychosomat. Med.* 65 461–470. 10.1097/01.PSY.0000035717.78650.A1 12764220

[B95] Stewart G. L. Courtright S. H. Manz C. C. (2019). Self-leadership: A paradoxical core of organizational behavior. *Annu. Rev. Organiz. Psychol. Organiz. Behav.* 6 47–67. 10.1146/annurev-orgpsych-012218-015130

[B96] Stone P. Luchetti R. (1985). Your office is where you are. *Harvard Bus. Rev.* 63 102–117. 10.5555/5080.5081

[B97] Tims M. Bakker A. B. Derks D. (2012). Development and validation of the job crafting scale. *J. Vocat. Behav.* 80 173–186. 10.1016/j.jvb.2011.05.009

[B98] Tims M. Derks D. Bakker A. B. (2016). Job crafting and its relationships with person-job fit and meaningfulness: A three-wave study. *J. Vocat. Behav.* 92 44–53. 10.1016/j.jvb.2015.11.007

[B99] Tucker L. R. Lewis C. (1973). A reliability coefficient for maximum likelihood factor analysis. *Psychometrika* 38 1–10. 10.1007/BF02291170

[B100] van der Voordt T. J. M. (2004). Productivity and employee satisfaction in flexible workplaces. *J. Corp. Real Estate* 6 133–148. 10.1108/14630010410812306

[B101] van Koetsveld R. Kamperman L. (2011). How flexible workplace strategies can be made successful at the operational level. *Corp. Real Estate J.* 1 303–319.

[B102] van Wingerden J. Bakker A. B. Derks D. (2017). Fostering employee well-being via a job crafting intervention. *J. Vocat. Behav.* 100 164–174. 10.1016/j.jvb.2017.03.008

[B103] Veldhoen Company (2021). *Activity based working.* Available Online at: https://www.veldhoencompany.com/en/activity-based-working/ (accessed September 14, 2021).

[B104] von Thiele Schwarz U. Richter A. Hasson H. (2018). *Getting Everyone on the Same Page. In Organizational Interventions for Health and Well-being.* Milton Park: Routledge, 42–67. 10.4324/9781315410494-3

[B105] Vos P. van der Voordt T. (2002). Tomorrow’s offices through today’s eyes: Effects of innovation in the working environment. *J. Corp. Real Estate* 4 48–65. 10.1108/14630010210811778

[B106] Wegman L. A. Hoffman B. J. Carter N. T. Twenge J. M. Guenole N. (2018). Placing job characteristics in context: cross-temporal meta-analysis of changes in job characteristics since 1975. *J. Manage.* 44 352–386. 10.1177/0149206316654545

[B107] Wilhelmsson M. (2016). *Varannan tycker arbetsmiljön försämras utan eget skrivbord (Every second person thinks the working environment is worsened without your own desk).* Nytorgsgatan: Finansliv.

[B108] Wohlers C. Hertel G. (2017). Choosing where to work at work – towards a theoretical model of benefits and risks of activity-based flexible offices. *Ergonomics* 60 467–486. 10.1080/00140139.2016.1188220 27167298

[B109] Wohlers C. Hartner-Tiefenthaler M. Hertel G. (2019). The relation between activity-based work environments and office workers’ job attitudes and vitality. *Environ. Behav.* 51 167–198. 10.1177/0013916517738078

[B110] Wrzesniewski A. Dutton J. E. (2001). Crafting a job: revisioning employees as active crafters of their work. *Acad. Manage. Rev.* 26 179–201. 10.5465/amr.2001.4378011

